# Branched-chain amino acids sustain pancreatic cancer growth by regulating lipid metabolism

**DOI:** 10.1038/s12276-019-0350-z

**Published:** 2019-11-29

**Authors:** Ji Hyeon Lee, Young-ra Cho, Ji Hye Kim, Jongwook Kim, Hae Yun Nam, Seong Who Kim, Jaekyoung Son

**Affiliations:** 1 Department of Biomedical Sciences, Asan Medical Center, AMIST, University of Ulsan College of Medicine, Seoul, 05505 South Korea; 2 Department of Biochemistry and Molecular Biology, Asan Medical Center, University of Ulsan College of Medicine, Seoul, 05505 South Korea

**Keywords:** Cancer, Cell growth

## Abstract

Branched-chain amino acid (BCAA) catabolism and high levels of enzymes in the BCAA metabolic pathway have recently been shown to be associated with cancer growth and survival. However, the precise roles of BCAA metabolism in cancer growth and survival remain largely unclear. Here, we found that BCAA metabolism has an important role in human pancreatic ductal adenocarcinoma (PDAC) growth by regulating lipogenesis. Compared with nontransformed human pancreatic ductal (HPDE) cells, PDAC cells exhibited significantly elevated BCAA uptake through solute carrier transporters, which were highly upregulated in pancreatic tumor tissues compared with normal tissues. Branched-chain amino-acid transaminase 2 (BCAT2) knockdown markedly impaired PDAC cell proliferation, but not HPDE cell proliferation, without significant alterations in glutamate or reactive oxygen species levels. Furthermore, PDAC cell proliferation, but not HPDE cell proliferation, was substantially inhibited upon knockdown of branched-chain α-keto acid dehydrogenase a (BCKDHA). Interestingly, BCKDHA knockdown had no significant effect on mitochondrial metabolism; that is, neither the level of tricarboxylic acid cycle intermediates nor the oxygen consumption rate was affected. However, BCKDHA knockdown significantly inhibited fatty-acid synthesis, indicating that PDAC cells may utilize BCAAs as a carbon source for fatty-acid biosynthesis. Overall, our findings show that the BCAA metabolic pathway may provide a novel therapeutic target for pancreatic cancer.

## Pancreatic cancer: targeting a link between amino acids and lipids

Essential nutrient molecules called branched-chain amino acids (BCAAs) sustain pancreatic cancer by supporting the formation of fatty ‘lipid’ compounds, offering possible new approaches for treatment. Jaekyoung Son and colleagues at the University of Ulsan College of Medicine in Seoul, South Korea, explored the role of BCAAs in the most common form of pancreatic cancer, pancreatic ductal adenocarcinoma. Effective treatments are urgently required as the disease is generally diagnosed too late for surgery, the most effective treatment, to be successful. The researchers found that human cancer cells exploit increased uptake of BCAAs as a source of carbon to build lipids. Disabling the genes for enzymes that convert BCAAs into lipids significantly and selectively reduced cancer cell growth. Drugs that interfere with key points in the over-active uptake of BCAA and conversion to lipids could be developed into new therapies.

## Introduction

Pancreatic cancer is a highly aggressive tumor with a poor prognosis^1^. Pancreatic ductal adenocarcinoma (PDAC) is the most common malignant pancreatic tumor, with an expected 5-year survival rate of ~8%^2^. PDAC is commonly detected after it has already spread beyond the pancreas because no signs or symptoms are apparent in the early stages, making it difficult to diagnose at an early stage^3^. Thus, only a small group of patients are candidates for surgery, which is the most effective treatment for PDAC. Unfortunately, it is predicted that PDAC will become the second leading cause of cancer-related death by 2020^4^. Thus, there is an urgent need for ideal therapeutic targets for pancreatic cancer treatment.

Compared with the levels in nonproliferating normal cells, the robust increases in the uptake of glucose and the production of lactate in cancer cells, known as the Warburg effect^5^, constitute the first hallmarks of cancer. Compared with normal differentiated cells, cancer cells have considerably different metabolic requirements that enable them to continuously grow. Thus, cancer cells must have altered metabolic pathways to obtain sufficient amounts of the metabolites required for high rates of proliferation^6^. Oncogene activation and/or epigenetic alterations drive changes in metabolism that support cell proliferation and survival under nutrient-replete conditions^7^. Thus, targeting the considerable metabolic differences between cancer cells and normal cells holds promise as an ideal strategy for cancer therapy^8^. Many studies have reported that abnormal levels of lipids are critical for the unlimited proliferation and the metastasis and invasion of cancer cells^9^. Highly proliferative cancer cells demand the rapid and sufficient synthesis of lipids for the generation of biological membranes. Thus, it is not surprising that the uptake of exogenous (or dietary) lipids is increased and endogenous lipid synthesis (lipogenesis) is induced as part of metabolic reprogramming in cancer cells. Lipid synthesis describes the processes by which nutrient-derived carbons are converted into fatty acids. Numerous studies have demonstrated that cancer cells show highly increased expression levels of genes involved in lipogenesis^10^. The expression levels of lipogenic enzymes, such as acetyl-CoA carboxylase (ACC) and fatty-acid synthase (FASN), and ATP-citrate lyase (ACLY) have been shown to be greatly increased in most tumors^11,12^. In addition, lipid metabolic reprogramming that promotes increased lipogenesis is associated with the abnormal development and progression of pancreatic adenocarcinoma^13^. Therefore, lipogenic enzymes such as FASN, ACLY, and ACC have been investigated as potential therapeutic targets for cancer treatment, and small compounds that inhibit these enzymes have shown therapeutic efficacy in various preclinical models of carcinogenesis^14–16^. However, substantial adverse side effects of targeting lipogenic enzymes have precluded their clinical development. Thus, there may be a need for new therapeutic targets to suppress lipogenesis.

Branched-chain amino acids (BCAAs; leucine, isoleucine, and valine) are essential amino acids that must be acquired from the diet. To support sustained biomass accumulation, cancer cells require increased levels of nutrients such as amino acids. Indeed, cancer cells have increased BCAAs uptake^17^, and BCAA aminotransferases (BCATs) that catalyze the first reaction in the catabolism of BCAAs are overexpressed in cancer cells^18,19^. In addition, BCAT2 protein levels are significantly increased in PDAC cells, and knockdown of BCAT2 in malic enzyme 2-deficient PDAC cells was shown to suppress the proliferation of these cells, indicating that BCAA metabolism plays an important role in PDAC cell proliferation^20^. However, the physiological roles of BCAA metabolism in cancer, particularly PDAC, remain unclear.

Here, we demonstrate that PDAC cells require BCAAs for proliferation as a carbon source for lipid biosynthesis. Knockdown of BCAT2 or BCKDHA, the key enzymes in BCAA catabolism, resulted in the significant suppression of PDAC cell proliferation, whereas human pancreatic ductal (HPDE) cell proliferation was not suppressed upon BCAT2 or BCKDHA knockdown. Interestingly, BCAT2 knockdown had no effect on either glutamate or reactive oxygen species (ROS) levels. Furthermore, neither the level of tricarboxylic acid (TCA) cycle intermediates nor the oxygen consumption rate was altered upon BCKDHA knockdown; however, BCKDHA knockdown led to a dramatic reduction in the levels of both fatty acids and triglycerides. Thus, our data suggest that BCAA metabolism may be an effective target for pancreatic cancer treatment.

## Materials and methods

### Cell culture

Cell lines were acquired from the American Type Culture Collection and were tested regularly for mycoplasma contamination. All cells were maintained at 37 °C in humidified air with 5% CO_2_ in Dulbecco’s modified Eagle’s medium (DMEM; Thermo Scientific, Waltham, MA, USA) supplemented with 10% fetal bovine serum, 100 U/mL penicillin, and 100 μg/mL streptomycin (Thermo Scientific, Waltham, MA, USA). The HPDE cells were cultured as described previously^21^.

### Reagents and antibodies

Antibodies against BCKDHA (sc-271538) and β-actin (sc-47778) were obtained from Santa Cruz Biotechnology (Santa Cruz, CA, USA). Antibodies against BCAT1 (12822), BCAT2 (9432), and ACLY (4332) were purchased from Cell Signaling Technology (Danvers, MA, USA). *N*-acetyl-l-cysteine (NAC; A9165) and palmitic acid (P0500) were obtained from Sigma-Aldrich (St. Louis, MO, USA). AdipoRed assay reagent (#PT-7009) was obtained from Lonza (Basel, Switzerland).

### Cell proliferation assay

Cells were seeded in 24-well plates (density: 2000 cells/well). The medium was not changed throughout the course of the experiment. At the indicated time intervals, the cells were fixed in 10% formalin and stained with 0.1% crystal violet. The dye was extracted with 10% acetic acid, and the relative proliferation level was determined according to the optical density measured at 595 nm.

### Colony formation assay

Cells were plated in six-well plates at 300 cells/well in 2 mL of medium. The medium was not changed throughout the course of the experiment. After 7–10 days, the cell colonies were fixed in 80% methanol and stained with 0.2% crystal violet.

### Detection of reactive oxygen species

To determine the levels of cytoplasmic ROS, the cells were incubated with 2′,7′-dichlorodihydrofluorescein diacetate (DCFDA, 5 μm; Invitrogen, Carlsbad, CA, USA) for 30 min. Excess DCFDA was removed by washing cells twice with phosphate-buffered saline (PBS) at room temperature; labeled cells were trypsinized, rinsed, and resuspended in PBS. The oxidation of DCFDA to the highly fluorescent 2′,7′-dichlorofluorescein, for which the intensity is proportionate to the level of ROS generation, was analyzed by flow cytometry. To analyze mitochondrial ROS, the cells were then incubated with 5 μm MitoSOX reagent (Thermo Scientific, Waltham, MA, USA) for 10 min at 37 °C and trypsinized, washed with PBS, and then resuspended in 200 μL of PBS. Stained cells were then quantified and analyzed on a flow cytometer (Beckman Coulter, Brea, CA, USA). The excitation wavelength was 510 nm, and the emission wavelength was 580 nm.

### Quantitation of intracellular ATP

Intracellular ATP concentrations were measured using an ATP colorimetric/fluorometric assay kit (BioVision Incorporated, Milpitas, CA, USA) in accordance with the manufacturer’s instructions. In brief, the cells were lysed in 100 μL of ATP assay buffer; 50 μL of the supernatant was collected and added to a 96-well plate. To each well, 50 μL of ATP assay buffer containing an ATP probe, ATP converter, and developer were added. Absorbance was measured at 570 nm.

### Transcript analysis of the clinical mRNA microarrays

Data on the transcription levels of transporters of BCAAs in normal and pancreatic cancer tissue were retrieved from the Oncomine platform (https://www.oncomine.org/), an online cancer microarray database. Using the default settings for various groups of filters, each query for accessing mRNA expression data was executed using threshold parameters of *p* value 1e–4, fold change 2, and gene ranking in the top 10%. Gene expression profiling interactive analysis (GEPIA) (http://gepia.cancer-pku.cn/) was used for analyzing RNA expression levels of the BCAA transporters and comparing them with those of normal and pancreatic cancer tissues.

### Metabolomics

Targeted liquid chromatography–mass spectrometry/mass spectrometry (LC-MS/MS) metabolomics analysis was performed as previously described^22^. In brief, the cells were grown to ~60% confluence in growth medium on 10 cm dishes. After 24 h, the cells were washed several times with phosphate-buffered saline and water, harvested using 1.4 mL of cold methanol/H_2_O (80/20, v/v), and lysed by vigorous vortex mixing; 100 μL of 5 μm internal standard was added. Metabolites were liquid–liquid extracted from the aqueous phase after adding chloroform. The aqueous phase was dried using vacuum centrifugation, and the sample was reconstituted with 50 μL of 50% methanol before the LC-MS/MS analysis.

### Oxygen consumption rate measurement

The oxygen consumption rate was measured using the Seahorse XF24 extracellular flux analyzer as previously described^22^. In brief, cells were plated in a 24-well Seahorse plate and cultured at 37 °C with 5% CO_2_. The medium was replaced the following day with unbuffered DMEM, and the cells were incubated at 37 °C without CO_2_ for 1 h. Oligomycin, FCCP, and rotenone were added to final concentrations of 2, 5, and 2 μm, respectively. For the measurement of fatty-acid oxidation, the oxygen consumption rate was assessed using the Seahorse XF24 extracellular flux analyzer, according to the manufacturer’s instructions. In brief, cells were seeded at a density of 5 × 10^4^ cells/well in 24-well plates and incubated for 24 h in growth medium. Endogenous substrates within the cells were depleted by replacing the culture medium with substrate-limiting medium (DMEM supplemented with 0.5 mm glucose, 1 mm GlutaMAX, 0.5 mm carnitine, and 1% fetal bovine serum) and incubating the cells for an additional 16 h. The medium was replaced with fatty-acid oxidation assay medium (111 mm NaCl, 4.7 mm KCl, 1.25 mm CaCl_2_, 2 mm MgSO_4_, and 1.2 mm NaH_2_PO_4_ supplemented with 2.5 mm glucose, 0.5 mm carnitine, and 5 mm HEPES; pH was adjusted to 7.4) and incubated for 45 min in a 37 °C incubator without CO_2_. Immediately prior to the assay, palmitate conjugated to bovine serum albumin (BSA) or control BSA was added to the appropriate wells, and the oxygen consumption rate (OCR) was measured at baseline and after the injection of oligomycin (0.5 μg/mL), FCCP (1 μm), or a combination of antimycin A (1 μm) and rotenone (1 μm). Fatty-acid oxidation was evaluated as the difference in maximum respiration after palmitate–BSA injection vs the baseline value.

### Glucose consumption and lactate production assay

Cells were plated in six-well plates (2 × 10^5^ cells/well). The medium was not changed throughout the course of the experiment and was collected at 24 h. Glucose and lactate concentrations in the medium were measured using a YSI 2300 STAT Plus glucose–lactate analyzer (YSI).

### Xenograft studies

To establish an intraperitoneal and intracranial xenograft mouse model, female severe combined immunodeficiency mice (18–20 g, 6 weeks of age) were purchased from Joong Ah Bio (Suwon, South Korea). All experimental procedures were conducted in accordance with a protocol approved by the Institutional Animal Care and Use Committee of Asan Institute for Life Sciences (protocol 2018-02-304). For subcutaneous xenografts, 8988 T cells were infected with lentiviral shRNA targeting BCKDHA (*n* = 2) or GFP (control hairpin, *n* = 1) and subjected to a short period of puromycin selection (2 μg/mL). A total of 1 × 10^6^ cells, suspended in 100 μL of Hank’s buffered saline solution, were injected subcutaneously into the lower flank of each mouse (four mice per group). Tumor length and width were measured twice weekly, and the volume was calculated according to the following formula: (length × width^2^)/2.

### Lentiviral-mediated shRNA targets

The following RNA interference (RNAi) Consortium clone IDs for shRNAs were used in this study: shBCAT2-1 (TRCN0000286203), shBCAT2-2 (TRCN0000286266), shBCKDHA-1 (TRCN0000028398), shBCKDHA-2 (TRCN0000028456), shACLY-1 (TRCN0000291817), and shACLY-2 (TRCN0000078285).

### Statistics

Data are presented as the mean ± standard deviation. All statistical comparisons were performed using unpaired Student’s *t* tests.

## Results

### PDAC cells display increased BCAA uptake

**Fig. 1:**
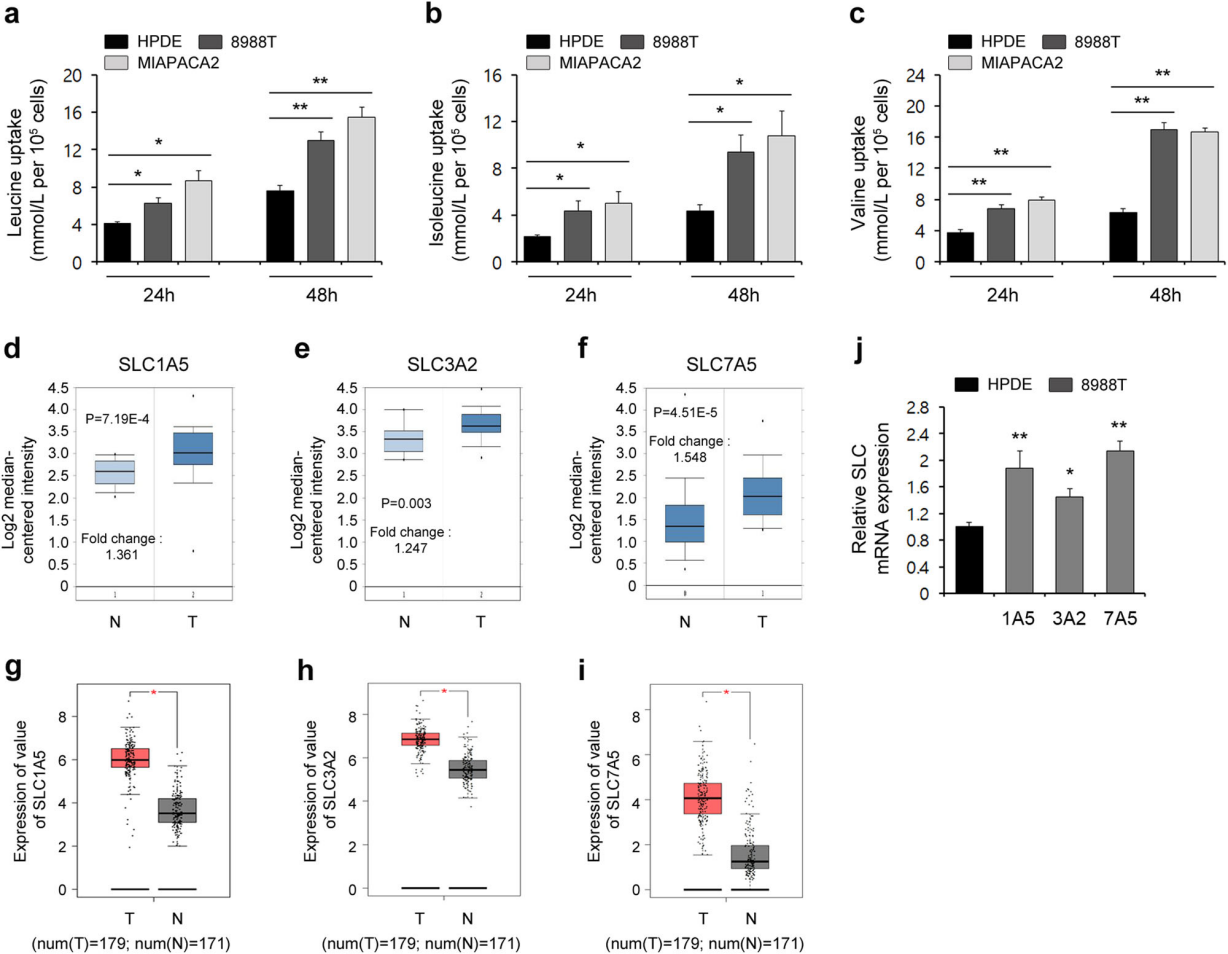
PDAC cells exhibit enhanced BCAA uptake. **a**–**c** BCAAs were analyzed at the indicated time points via LC-MS/MS. **d**–**f** Box plots derived from gene expression data in Oncomine comparing the expression levels of SLC transporter genes in normal tissue samples (left plot) and in pancreatic cancer samples (right plot). **g**–**i** The expression levels of SLC transporter genes were detected in 171 pancreatic cancer tissues (T) and 179 normal tissues (N) from the GEPIA database. **j** The expression levels of SLC transporter genes were determined by quantitative RT-PCR. Error bars represent the s.d. of triplicate wells from a representative experiment. *p < 0.05; **p < 0.01.

As the role of BCAAs in tumor growth is complex and depends on the tumor type, we explored the importance of BCAAs in PDAC growth. To this end, we first tested whether PDAC cells exhibit an increase in BCAA uptake. As shown in Fig. 1a, PDAC cells, including 8988 T and MIAPACA2 cells, exhibited significantly elevated leucine uptake compared with that of the HPDE cells, and the relative changes in leucine uptake increased gradually over time. Consistent with this finding, the uptake of other BCAAs, namely, isoleucine and valine, by the PDAC cells was greatly increased compared with that of the HPDE cells (Fig. 1b, c). Given that several solute carrier (SLC) transporters (Slc1a5, Slc3a2, and Slc7a5) are known to mediate BCAA transport^23–25^, we speculate that the expression of these SLC transporters might be elevated in PDAC cells. First, we analyzed the expression of these SLC transporters in human pancreatic cancer using the Oncomine database (available at http://www.oncomine.org). The gene expression levels of these SLC transporters were higher in pancreatic cancer samples than in normal tissue samples (Fig. 1d–f). Furthermore, the gene expression levels of the SLC transporters was validated using the GEPIA database (available at http://gepia.cancer-pku.cn/) and were observed to be higher in 179 pancreatic cancer tissues than they were in 171 normal tissues (Fig. 1g–i). Consistent with these findings, the expression levels of the SLC transporters at the transcriptional level were increased in the PDAC cells compared with those in the HPDE cells (Fig. 1j). These findings demonstrate that PDAC cells exhibit elevated levels of BCAA uptake.

### BCAT2 is critical for PDAC growth

**Fig. 2:**
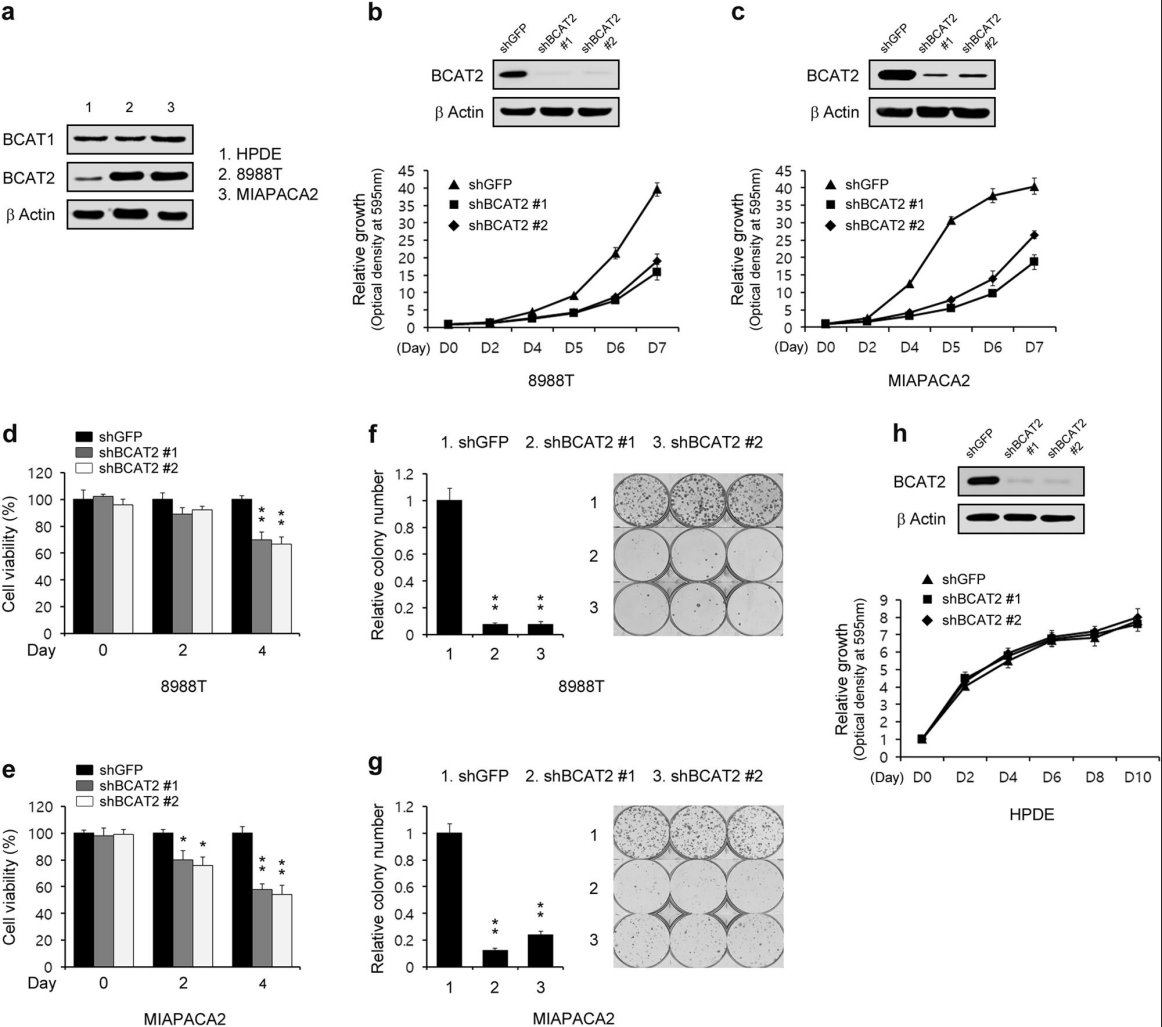
Pancreatic cancer cells require BCAT2 to proliferate. **a** Immunoblots of BCAT1 and BCAT2 expression in HPDE and PDAC cells. **b**, **c** PDAC cells expressing control shRNA (shGFP) or one of the two independent shRNAs to downregulate BCAT2 were assayed for cell proliferation. Western blot confirmed the knockdown of BCAT2 expression. **d**, **e** PDAC cells expressing control shRNA (shGFP) or one of the two independent shRNAs to downregulate BCAT2 were assayed for cell viability. **f**, **g** Relative clonogenic growth levels of PDAC cells expressing control shRNA (shGFP) or one of the two independent shRNAs targeting BCAT2. **h** HPDE cells expressing control shRNA (shGFP) or one of the two independent shRNAs to downregulate BCAT2 were assayed for cell proliferation. Western blot confirmed the knockdown of BCAT2 expression. Error bars represent the s.d. of triplicate wells from a representative experiment. *p < 0.05; **p < 0.01.

One of the metabolic changes exhibited by tumor cells is an increase in glucose uptake; this metabolic change causes cancer cells to become highly dependent on glucose for proliferation. Given that PDAC cells displayed increased BCAA uptake, we examined the importance of BCAA metabolism in PDAC cell proliferation. BCAT2 is a mitochondrial isoform required for the first step in BCAA catabolism. Notably, the BCAT2 activity in the exocrine pancreas is one of the highest levels of among all tissues and organs^26^. The PDAC cells exhibited increased levels of BCAT2 expression, but not BCAT1 expression, compared with that of the HPDE cells (Fig. 2a), a finding that is consistent with the recent work showing that BCAT2 protein levels are significantly increased in PDAC cells^20^. We next tested the importance of BCAT2 in PDAC cell proliferation. To explore the effect of BCAT2 on PDAC cell proliferation, we first downregulated BCAT2 activity using RNAi. As shown in Fig. 2b, c, BCAT2 knockdown markedly reduced PDAC cell proliferation. To confirm that BCAT2 knockdown inhibited PDAC cell proliferation, we next performed an assay of cell viability upon BCAT2 knockdown. Consistent with the growth curve data, BCAT2 knockdown resulted in a profound reduction in the proliferation of PDAC cells (Fig. 2d, e). In addition, PDAC cell colony formation was markedly inhibited upon BCAT2 knockdown (Fig. 2f, g). In contrast to their importance in PDAC cells, BCAT2 appears to be dispensable in normal cells. BCAT2 knockdown in HPDE cells had modest effects on proliferation (Fig. 2h). These findings indicate that BCAT2 may be critical only for PDAC cell proliferation.

### BCAT2 suppression does not affect redox homeostasis

**Fig. 3:**
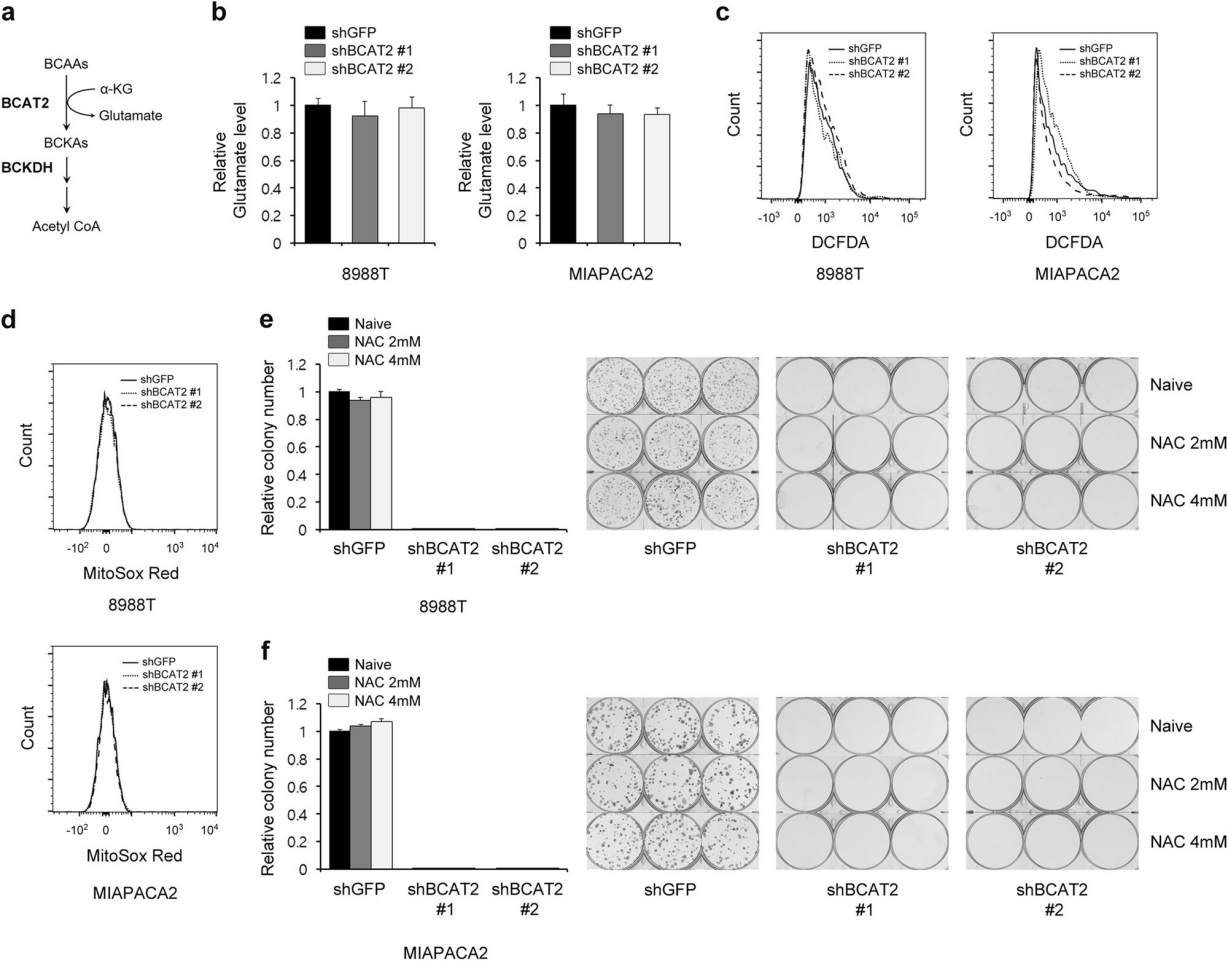
BCAT2 has no effect on redox homeostasis. **a** Schematic depiction of the reactions that convert BCAAs into acetyl-CoA. **b** Relative glutamate levels of PDAC cells expressing control shRNA (shGFP) or shRNA targeting BCAT2. **c**, **d** PDAC cells expressing control shRNA (shGFP) or shRNA targeting BCAT2 were subjected to the DCFDA assay **c** or MitoSOX Red assay **d**. **e**, **f** Relative clonogenic growth of PDAC cells expressing control shRNA (shGFP) or shRNA targeting BCAT2 with or without NAC (4 mm). Error bars represent the s.d. of triplicate wells from a representative experiment.

BCAT2, also known as mitochondrial BCAT, facilitates the transfer of an α-amino group from the BCAAs to α-ketoglutarate to generate glutamate and the respective branched-chain α-keto acid (Fig. 3a). It has been reported that BCAT2 inhibition suppresses glutamate biosynthesis and redox homeostasis in glialoma^27^. To investigate the role of BCAT2 in PDAC cell proliferation, we first measured the glutamate levels upon BCAT2 knockdown. As shown in Fig. 3b, BCAT2 knockdown had no significant effect on glutamate levels. Moreover, neither cytoplasmic nor mitochondrial ROS levels were altered upon BCAT2 knockdown (Fig. 3c, d). In addition, supplementation with NAC did not restore the cell proliferation that had been inhibited by BCAT2 knockdown (Fig. 3e, f). These findings suggest that BCAT2 has no critical role in glutamate biosynthesis or redox homeostasis in PDAC cells.

### BCKDHA knockdown suppresses PDAC cell proliferation

**Fig. 4:**
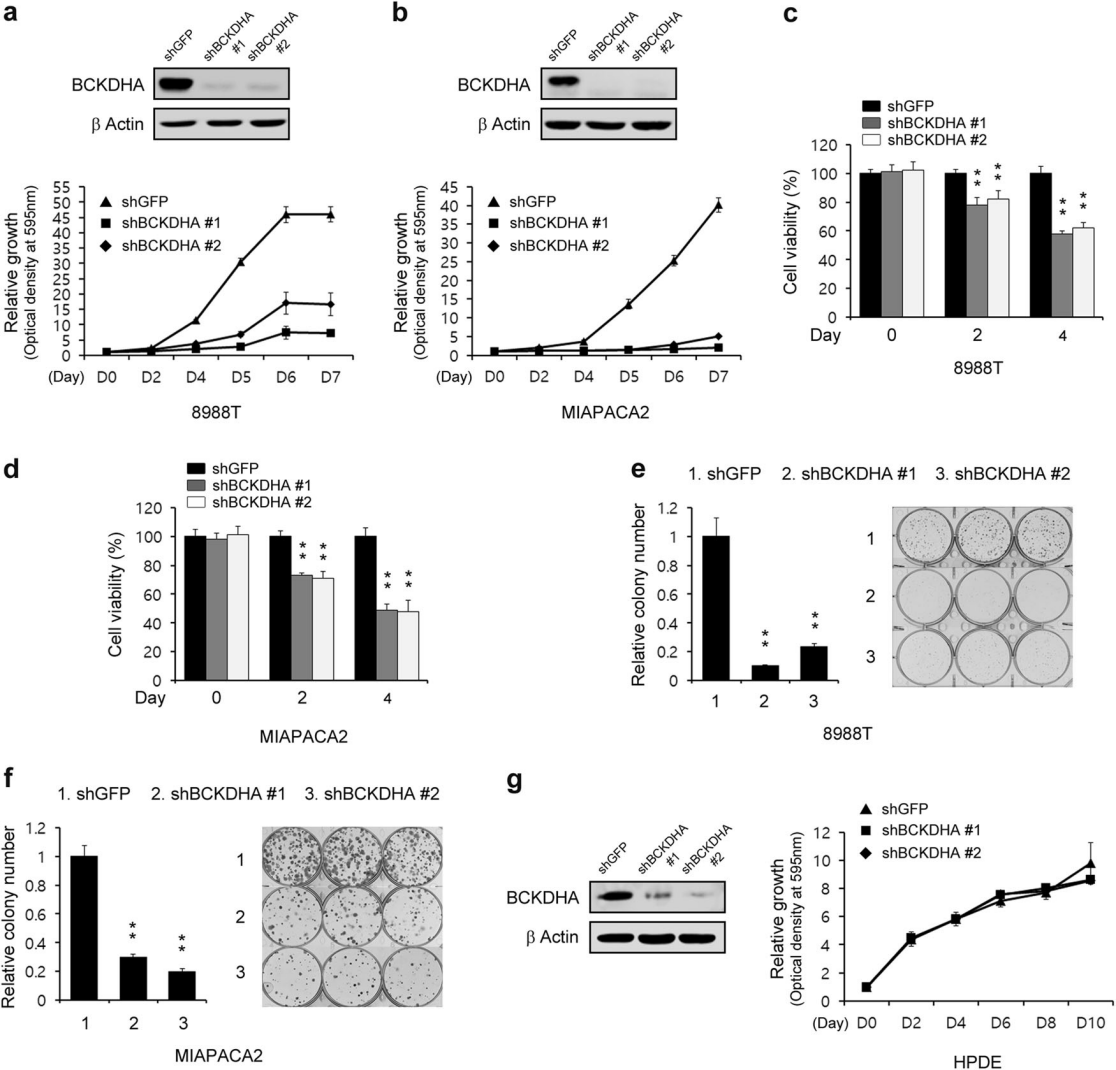
Pancreatic cancers require BCKDHA for proliferation. **a**, **b** PDAC cells expressing control shRNA (shGFP) or shRNA targeting BCKDHA were assayed for cell proliferation. **c**, **d** PDAC cells expressing control shRNA (shGFP) or shRNA targeting BCKDHA were assayed for cell viability. **e**, **f** Relative clonogenic growth of PDAC cells expressing control shRNA (shGFP) or shRNA targeting BCKDHA. **g** HPDE cells expressing control shRNA (shGFP) or one of the two independent shRNAs targeting BCKDHA were assayed for cell proliferation. Western blot confirmed the knockdown of BCKDHA expression. Error bars represent the s.d. of triplicate wells from a representative experiment. *p < 0.05; **p < 0.01.

To explore the roles of BCAA metabolism in PDAC proliferation, we next tested the effect of BCKDHA knockdown on PDAC cell proliferation. BCKDHA, the enzyme catalyst for the second step of the BCAA catabolic pathway, which has a central role in the regulation of BCAA catabolism, has been shown to promote tumorigenesis that leads to colorectal cancer^28^. Consistent with this finding, BCKDHA knockdown significantly reduced PDAC cell proliferation (Fig. 4a, b). To further confirm the effect of BCKDHA knockdown on PDAC cell proliferation, we next assayed cell viability upon BCKDHA knockdown. Consistent with the proliferation curve, BCKDHA knockdown led to a profound reduction in the proliferation of PDAC cells (Fig. 4c, d). In addition, PDAC cell colony formation was dramatically inhibited upon BCKDHA knockdown (Fig. 4e, f). Interestingly, the proliferation of HPDE cells, in contrast to that of the PDAC cells, was not impaired upon BCKDHA knockdown (Fig. 4g). These findings indicate that PDAC, but not normal cells, may rely on BCAA metabolism to support growth.

### Lipogenesis requires BCAA metabolism

**Fig. 5:**
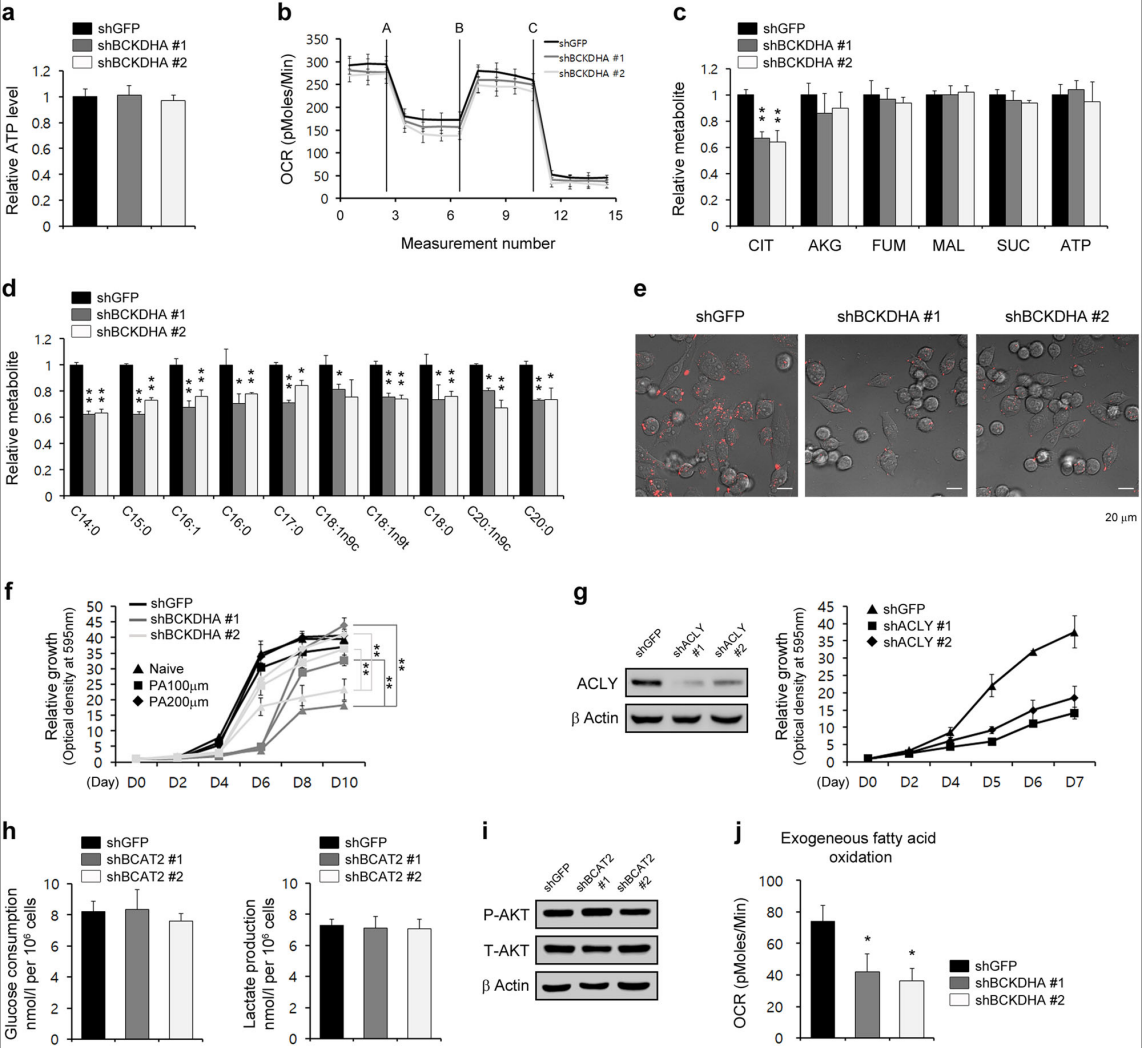
BCAA metabolism is critical for fatty-acid biosynthesis. **a** 8988 T cells expressing control shRNA (shGFP) or shRNA targeting BCKDHA were assayed for intracellular ATP levels. **b** Oxygen consumption rates in 8988 T cells expressing control shRNA (shGFP) or BCKDHA shRNA (shBCKDHA) were measured with an extracellular flux analyzer. Cells were sequentially treated with oligomycin (2 μm), FCCP (5 μm), and rotenone (2 μm). **c**, **d** TCA metabolite and free fatty-acid pools in 8988 T cells expressing control shRNA (shGFP) or BCKDHA shRNA (shBCKDHA) were analyzed via LC-MS/MS: **c** TCA metabolite pools and **d** free fatty-acid pools. Error bars represent the s.d. of triplicate wells from a representative experiment. **e** 8988 T cells expressing control shRNA (shGFP) or shRNA targeting BCKDHA were assayed for intracellular triglyceride levels with AdipoRed staining. **f** 8988 T cells expressing control shRNA (shGFP) or shRNA targeting BCKDHA were plated in complete medium that was supplemented the following day with 100 or 200 μm palmitic acid and assayed for cell proliferation. Error bars represent the s.d. of triplicate wells from a representative experiment. **g** 8988 T cells expressing control shRNA (shGFP) or shRNA targeting ACLY were assayed for cell proliferation. Western blot confirmed the knockdown of ACLY expression. **h** 8988 T cells expressing control shRNA (shGFP) or shRNA targeting BCAT2 were plated in complete media; glucose uptake and lactate production were measured using a YSI 2300 STAT Plus glucose–lactate analyzer (YSI). The error bars represent the s.d. of triplicate wells from a representative experiment. **i** 8988 T cells expressing control shRNA (shGFP) or shRNA targeting BCAT2 were immunoblotted with the indicated antibodies. **j** To assess the extent of fatty-acid oxidation, the oxygen consumption rates in 8988 T cells expressing control shRNA (shGFP) or shRNA targeting BCKDHA were measured using an extracellular flux analyzer. Error bars represent the s.d. of triplicate wells from a representative experiment. PA, palmitic acid. *p < 0.05; **p < 0.01.

Given that BCAAs fuel the TCA cycle by transferring respective amino groups to the TCA cycle^25^, we first examined ATP levels upon BCKDHA knockdown to investigate the functional role of BCKDHA in TCA metabolism. As shown in Fig. 5a, the ATP levels were not highly affected upon BCKDHA knockdown. In addition, BCKDHA knockdown had no significant effect on oxygen consumption rates (Fig. 5b). To validate the effect of BCAA catabolism on the levels of TCA cycle intermediates, we performed a metabolic analysis. The levels of TCA cycle intermediates were not changed upon BCKDHA knockdown (Fig. 5c). With a variety of functions, BCAA metabolism contributes to fatty-acid biosynthesis by providing a carbon source to generate fatty acids^29^. Indeed, the levels of free fatty acids were significantly reduced upon knockdown of either BCKDHA or BCAT2 (Fig. 5d and Supplementary Fig. S1a), and consistent with this finding, knockdown of either BCKDHA or BCAT2 had a substantial inhibitory effect on triglyceride accumulation (Fig. 5e and Supplementary Fig. S1b), indicating that BCAAs may be essential for lipid biosynthesis. To confirm the importance of BCAAs in lipid biosynthesis, we attempted to rescue the inhibition of cell proliferation by BCKDHA knockdown via supplementation of the culture medium with palmitic acid. We found that palmitic acid supplementation significantly rescued the cells from BCKDHA knockdown-mediated inhibition of proliferation (Fig. 5f). In addition, consistent with the effects of BCAT2 and BCKDHA knockdown on PDAC cell proliferation, knockdown of ACLY, a key enzyme in fatty-acid biosynthesis, dramatically suppressed PDAC cell proliferation (Fig. 5g).

It has been reported that BCAAs regulate glucose metabolism through the PI3K–Akt pathway^30^. Thus, we investigated whether the suppression of PDAC cell proliferation caused by the disruption of BCAA metabolism can be attributed to the inhibition of glucose metabolism. As shown in Fig. 5h, i, neither glucose uptake nor lactate production was changed upon BCAT2 knockdown. Moreover, BCAT2 knockdown had no effect on AKT phosphorylation. In addition, BCAA treatment activated fatty-acid oxidation^31^. Thus, we assessed the possibility that BCKDHA knockdown may increase the levels of the BCAAs, which in turn would reduce the levels of free fatty acids through fatty-acid oxidation. As shown in Fig. 5j, fatty-acid oxidation decreased upon BCKDHA knockdown. Taken together, these findings suggest that PDAC cells may utilize BCAAs as carbon sources for fatty-acid biosynthesis.

### BCKDHA knockdown inhibits tumor growth

**Fig. 6:**
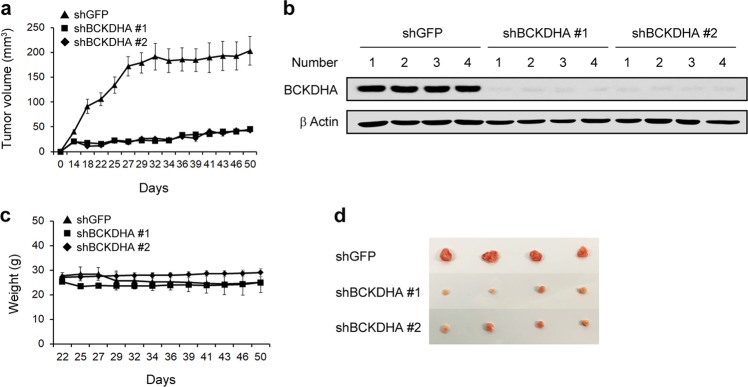
Effect of BCKDHA on tumor growth. **a** Xenograft growth of 8988 T cells expressing control shRNA (shGFP) or shRNA targeting BCKDHA (1 and 2) in mice. Error bars represent s.e.m. (*n* = 4). **b** Xenograft tumors were collected, and tissue lysates were blotted for BCKDHA detection. **c** Record of body weights of the mice. **d** Representative images of xenograft tumors obtained from the mice.

To determine the effect of BCKDHA on tumor growth, we subcutaneously injected 8988 T cells expressing control shRNA (shGFP) or BCKDHA shRNA (shBCKDHA) into mice to construct a xenograft mouse model. As shown in Fig. 6a, BCKDHA knockdown had a significant inhibitory effect on tumor growth. Xenograft tumors generated from human PDAC cells expressing BCKDHA shRNA were subjected to Western blot for BCKDHA (Fig. 6b). Moreover, BCKDHA knockdown did not significantly decrease the body weight of the mice, indicating that downregulating BCKDHA did not induce obvious toxicity (Fig. 6c). We also observed that BCKDHA knockdown significantly reduced the size of the xenograft tumors (Fig. 6d). These observations demonstrated that BCKDHA knockdown leads to the significant inhibition of tumor growth in vivo.

## Discussion

The results obtained in this study demonstrate that BCAA metabolism provides a carbon source for fatty-acid biosynthesis, which sustains pancreatic cancer cell proliferation. Knockdown of key enzymes, including BCAT2 and BCKDHA, substantially inhibits PDAC cell proliferation, and interestingly, suppression of BCAA metabolism leads to a significant reduction in fatty-acid levels.

BCAAs have unique properties with various physiological and metabolic roles. Thus, they have important metabolic functions in the regulation of energy homeostasis, nutrition metabolism, gut health, immunity, and cancer^32^. The reprogramming of metabolic pathways is a common hallmark of cancer. One of the outcomes of such metabolic reprogramming is an increase in the amounts of basic cellular building blocks, such as carbohydrates and amino acids, which are necessary to support unlimited cell proliferation^33^. Indeed, many tumors exhibit a significant increase in BCAA uptake compared with adjacent normal tissue, and the inhibition of BCAA catabolism has been shown to suppress tumor growth^17,34^. Consistent with these findings, our results show that PDAC cells have increased BCAAs uptake compared with HPDE cells (Fig. 1a–c). Thus, it is possible that the catabolism of these elevated levels of BCAA may play a critical role in tumor growth. Indeed, genetic disruption of the key enzymes involved in BCAA catabolism inhibits the growth of tumors^17–19,28^. Our data also showed that knockdown of either BCAT2 or BCKDHA significantly inhibited PDAC cell proliferation but not HPDE cell proliferation (Figs. 2 and 4).

Previous studies have suggested that BCAT2 supports glioma growth by maintaining glutamate biosynthesis and redox homeostasis^27^. However, our results revealed that there is no significant change in either glutamate levels or redox homeostasis upon BCAT2 knockdown. PDAC cells have distinct metabolism for both the uptake and utilization of glutamine compared with the cells in other Kras-driven tumors. Macropinocytosis has an essential role in providing glutamine in PDAC^35^, and PDAC cells depend on the transaminase GOT1 for glutamine metabolism, which is critical for redox balance^36^. Thus, we believe that BCAT2 may have other roles in PDAC cell metabolism in addition to maintaining glutamate levels and redox homeostasis. Recent work reported by DePinho and colleagues and our results showed that PDAC cells have increased levels of BCAT2 expression but not of BCAT1 expression. However, further work must be performed to determine why BCAT2, but not BCAT1, is overexpressed and essential in PDAC cells.

Consistent with recent studies implicating BCAA metabolism in lipid metabolism^37,38^, we observed that inhibition of BCAA metabolism suppressed lipid metabolism. In general, glucose, glutamine, and other substrates are the main sources of cytosolic acetyl-CoA, which in turn is the two-carbon donor for fatty-acid synthesis^39^. Compared with glucose, glutamine is utilized as the main carbon source for the TCA cycle in PDAC cells^40^. Glutamine contributes to citrate and lipid metabolism through TCA cycle reversal via reductive carboxylation. However, in contrast to other cancer cells that utilize glutamine to fuel the TCA cycle, in PDAC cells, glutamine is used principally to maintain the redox balance through transaminase-mediated glutamine metabolism^36^. Thus, previous studies and our data suggest that PDAC cells require BCAAs as alternative carbon sources to generate fatty acids.

BCKDHA has been shown to promote the tumorigenesis that leads to colorectal cancer^28^. It is a key enzyme in the BCAA catabolic pathway that generates acetyl-CoA from BCAAs fuel the TCA cycle^25^. In contrast to the levels in other tumor cells, in PDAC cells, carbon from the BCAAs of the TCA cycle metabolites was not detected^20^. Consistent with this finding, our data revealed that the inhibition of BCAA catabolism caused by the suppression of BCKDHA activity had no significant effect on the level of TCA cycle metabolites (Fig. 5c), suggesting that BCAAs are not a major carbon source for the TCA cycle in PDAC cells. Interestingly, among the TCA cycle intermediates, only the levels of citrate were decreased upon BCKDHA knockdown (Fig. 5c). Citrate generated in the TCA cycle and subsequently translocated to the cytosol serves as a carbon source for fatty-acid biosynthesis. Enhanced lipogenesis is one of the most important metabolic hallmarks of cancer cells^41^. Specifically, cancer cells induce metabolic alterations in lipid metabolism to enable unlimited cell proliferation and survival^42^. Indeed, we observed that either BCKDHA or BCAT2 knockdown led to both a profound reduction in the levels of free fatty acids (Fig. 5d and Supplementary Fig. S1a) and a significantly decreased level of triglyceride accumulation (Fig. 5e and Supplementary Fig. S1b). In addition, we found that fatty-acid oxidation was reduced upon BCKDHA knockdown (Fig. 5j), and this reduction may have been caused by the utilization of lower levels of fatty acids (observed after BCKDHA knockdown) for functions such as membrane generation and protein modification rather than as fuel for the TCA cycle. Thus, our findings suggest that BCAAs are utilized as carbon sources for fatty-acid biosynthesis in PDAC cells.

In an important contrast to HPDE cells, PDAC cells are highly dependent on the BCAA catabolic pathway. Genetic inhibition of any enzyme in this pathway results in the pronounced suppression of PDAC cell proliferation in vitro and in vivo. Given our findings, disruption of the BCAA catabolic pathway may be an exploitable therapeutic target for pancreatic cancer therapy.

## Supplementary information

Supplementary information
