# Supplementary material for: Branched-chain amino acids sustain pancreatic cancer growth by regulating lipid metabolism
Source: Exp Mol Med. 2019 Nov 29;51(11):146. doi: 10.1038/s12276-019-0350-z (PMC6884453; doi:10.1038/s12276-019-0350-z)
Supplement: Supplementary file 1 — Supplementary information [file 12276_2019_350_MOESM1_ESM.pdf]

Supplemental Information

Branched-chain amino acids sustain pancreatic cancer growth by regulating lipid metabolism

Ji Hyeon Lee[1,*], Young-ra Cho[1,*], Ji Hye Kim[1], Jongwook Kim[1], Hae Yun Nam[2], Seong Who Kim[2], Jaekyoung Son[1]

[1]Department of Biomedical Sciences, Asan Medical Center, AMIST, University of Ulsan College of Medicine, Seoul 05505, South Korea

[2]Department of Biochemistry and Molecular Biology, Asan Medical Center, University of Ulsan College of Medicine, Seoul 05505, South Korea

*These authors contributed equally to this work.

Correspondence: Jaekyoung Son,

Department of Biomedical Sciences, University of Ulsan College of Medicine,

88, Olympic-ro 43-gil, Songpa-gu, Seoul 05505, South Korea

Phone: 82-2-3010-4184; Fax: 82-2-3010-2097; E-mail: jaekson@amc.seoul.kr

Supplementary Figure 1

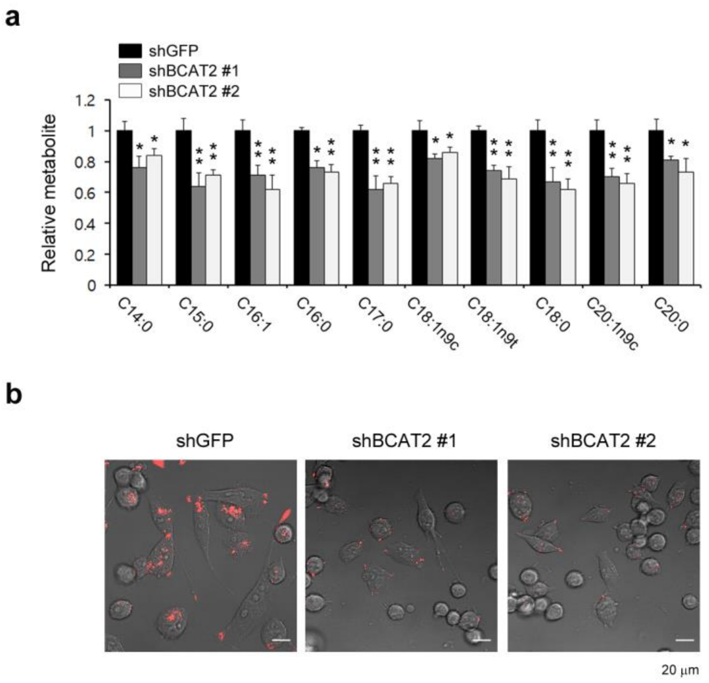

Supplementary Figure 1. BCAT2 knockdown results in significant reductions of levels of both fatty acids and triglycerides. **a** Free fatty acid pools in 8988T cells expressing control (shGFP) or shRNAs targeting BCAT2 were analyzed via LC-MS/MS. **b** 8988T cells expressing control (shGFP) or shRNAs targeting BCAT2 were assayed for intracellular triglyceride levels with Adipo Red staining. Error bars represent the s.d. of triplicate wells from a representative experiment. *, $p < 0.05$; **, $p < 0.01$.
